# Lectotypification of six names in the genus *Elleanthus* (Orchidaceae) described from J. J. Linden’s collection

**DOI:** 10.3897/phytokeys.182.68782

**Published:** 2021-09-24

**Authors:** Magdalena Dudek, Dariusz L. Szlachetko

**Affiliations:** 1 Department of Plant Taxonomy and Nature Conservation, Faculty of Biology, University of Gdańsk, Wita Stwosza 59, PL-80-308 Gdańsk, Poland University of Gdańsk Gdansk Poland

**Keywords:** *
Elleanthus
*, *
Evelyna
*, Jean Jules Linden, lectotype, nomenclature, syntype

## Abstract

The lectotypification of six names of species, originally described as *Evelyna* Lind. (Orchidaceae), based on collections of Jean Jules Linden from locations that are currently in Venezuela and Colombia, is proposed. We also provide the number and location of duplicates of the type material.

## Introduction

The Neotropical orchid genus *Elleanthus* was proposed by Czech botanist Carl Borivoj Presl in 1827 (Presl 1927). As the genus has never been comprehensively taxonomically revised, we can assume that it currently comprises over 120 species (Dodson and Luer 2010) which are widely distributed throughout the tropical and subtropical zones of the New World (Pridgeon et al. 2005). We can also assume that a significant number of taxa are still awaiting their formal description. The works by Garay (1978), Dodson (1998) and Dodson and Luer (2010) – the only partial revisions of the genus – concern solely the species growing in Ecuador, which is a comparatively minor part of the whole range of the genus. The difficulties in studying *Elleanthus* likely ensue from the high morphological diversity of these plants, especially the structure of the flowers (e.g. shape, size and texture of floral bracts, shape and placement callus on the lip, size and shape of lip, structure of gynostemium), but also the vegetative parts (e.g. type of stem, size, shape and texture blade of leaf). The strong floral polymorphism is also the reason for many systematic ambiguities with reference to the genus.

Over the years, researchers have described a few similar genera, *Evelyna* Poepp. & Endl., *Adeneleterophora* Barb.Rodr. and *Epilyna* Schltr., which have been considered synonymous with *Elleanthus* (Reichenbach 1861; Dressler 1981, 1993; Szlachetko 1995; Pridgeon et al. 2005). The first of them was characterised by flowers densely packed in capitate inflorescence, equal sepals, a sub-rounded lip that is saccate at the base and features two calli and a semi-terete, naked gynostemium. It originally embraced five species (*Evelynaaurea*, *E.capitata*, *E.gaminifolia*, *E.oligantha* and *E.strobilifera*) which were described by Poepping and Endlicher (1835).

Many orchid species were described in the 19^th^ century without indicating any type material, or information about the location of the reference collection. The examination of any original material cited in the protologue is a remarkable step in taxonomic work. Our studies towards revising the genus *Elleanthus* sensu lato has revealed specimens, belonging to the Jean Jules Linden’s gatherings, where Lindley (1846) described seven species of *Evelyna* (*E.bractescens*, *E.columnaris*, *E.ensata*, *E.flavescens*, *E.furfuracea*, *E.kermesina* and *E.lupulina*). We also found the original material for one species (*Evelynacoriifolia*), which was described by Reichenbach (1852). Though they do not have a capitate inflorescence, they were placed in the genus proposed by Poeppig and Endlicher in 1835. However, several years later, Reichenbach (1861) came to the conclusion that they are congeneric and transferred species of this genus to *Elleanthus*.

Jean Jules Linden was a Belgian botanist and was particularly fond of orchids. His first scientific expedition to Brazil lasted less than two years (September 1835–March 1837). Nicolas Funck, Auguste Ghiesbreght and he collected and brought back to Europe a large collection of plants and animals. During the following expedition (September 1837–December 1840) to Cuba and Mexico, he focused mainly on observing the habitat of orchids and collected them for breeding purposes and for adding to European herbaria. He was the first botanist whose findings revolutionised the cultivation of orchids in Europe (Ceulemans and Braem 2006). His other trip to Venezuela and Colombia (1841–1844) resulted in gathering a variety of orchids species that Lindley (1846) described in Orchidaceae Lindenianae including *Evelyna* species. Lindley mentioned in his work that Linden had made partial contributions to some English institutions. However, the most of plants collected by Linden were cultivated in Brussels, Ghent and Paris (Ceulemans and Braem 2006), and during our studies we found Linden’s collection at P and BR Herbaria.

Currently, we are carrying out a revision of *Elleanthus* sensu lato (in preparation), which is proving that some names of species are lacking type. In order to stabilise the nomenclature of these species, they require typification. Therefore, in this paper, a lectotypification of the names of species of *Elleanthus* described from Linden’s gathering is proposed. For one name, we could not indicate a lectotype and this is widely discussed.

## Typifications

### Elleanthus bractescens

Taxon classificationPlantaeAsparagalesOrchidaceae

(Lindl.) Rchb.f., Annales Botanices Systematicae 6: 479. 1861.

85A81AED-3D71-5F45-B274-A07C5A7651F8

Basionym. Evelynabractescens Lindl., Orchidaceae Lindenianae 10,11, no 59. 1846. Type: Venezuela, Merida, “on the stunted trees skirting the paramos of the Province Merida at the height of 8000 to 10 000 feet [2438–3480 m], July”, *Linden 2215*.

#### Note.

In the protologue of *Evelynabractescens*, Lindley (1846:11) included just a short note concerning the locality and collector of the reference specimen. In his Orchids of Peru, Schweinfurth (1958) noted that the type of *Evelynabractesces* is stored in Lindley Herbarium at Kew. We were, however, unable to locate there any sheets labelled *Evelynabractescens*. Interestingly, instead we found a specimen of *Maxillarialongissima* Lindl. collected by Linden in Merida Province and numbered *2215* (K-000779830). This sheet has the original label with Linden’s handwriting. Additionally, we checked a protologue of *M.longissima* and it appeared that this species was described, based on the gathering named *Linden 2215* [coll. orig. ‘Forests of Merida, at the height of 6000 feet; July (No. 2215)’] (Lindley 1846).

**Figure 1.**
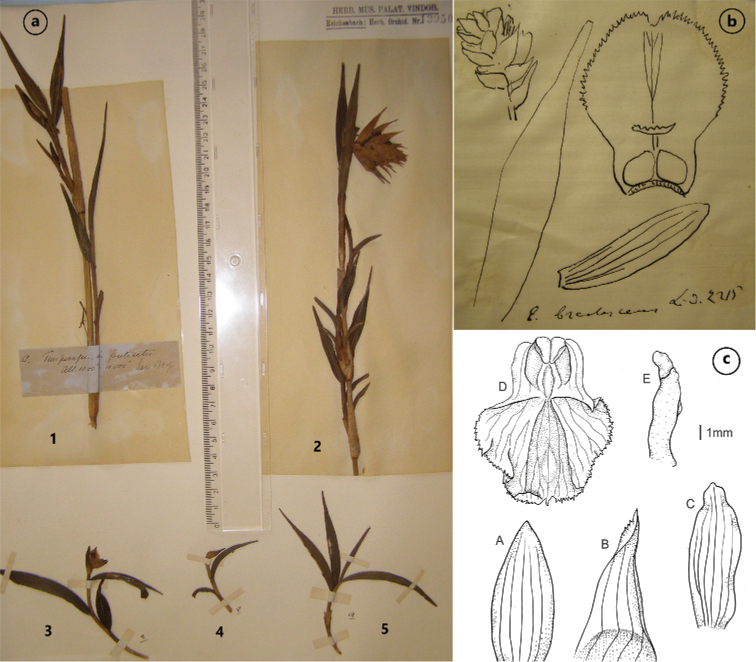
*Evelynabractescens* Lindl. **a** specimen *Linden 2215* at the Naturhistorisches Museum, Vienna (W-R13950) **b** Reichenbach’s drawing **c** based on Szlachetko’s drawing of the Fragment 2 of original material (Baranow): A – dorsal sepal, B – lateral sepal, C – petal, D – lip, E – gynostemium.

During our research in herbaria, we located only a single *Linden 2215* collection corresponding to *Evelynabractescens*. It is deposited in the Naturhistorisches Museum in Vienna (W-R13950). This sheet includes five plant fragments and Reichenbach’s drawing, signed as *E.bractescens* and *Lin. 2215* (Fig. 1a, b). The first fragment, including a sterile stem with leaves, but no flowers, has a label with the locality recorded as ‘Tungurahura [Ecuador] in fruticetis, alt. 1000’–11000’, Jan. 1859’ (Fig. 1a). Therefore, it is not mentioned in the protologue. Fragments 3, 4 and 5 (Fig. 1a) have no labels; they comprise only a very small part of the stem and a few leaves, hence they are indeterminable. However, Fragment 2 (Fig. 1a) is the only one to include an inflorescence with flowers. After a detailed analysis of the flower’s parts and a comparison with the original description, it seems that Fragment 2 corresponds to the description of *Evelynabractescens*. However, it has no locality data to confirm the information contained in the protologue. Only the note on the drawing suggests Linden’s gathering. We can suspect that Reichenbach prepared this drawing (Fig. 1b), based on Fragment 2. Unfortunately, we are unable to clearly indicate where the type collection is stored, so we decided not to lectotypify this name.

### Elleanthus columnaris

Taxon classificationPlantaeAsparagalesOrchidaceae

(Lindl.) Rchb.f., Annales Botanices Systematicae 6: 483. 1861.

4B0CCADB-E56D-50D6-ABD8-CCB2CBD5C2DB

Basionym: Evelynacolumnaris Lindl., Orchidaceae Lindenianae 11. no. 62. 1846. Type: Venezuela, Trujillo, “Agua de Obispo and Sierra Nevada, at high of 9000 feet [2743 m], May to August”, *Linden 620*; Lectotype (designated here): P (P00389742), drawing of the lectotype (K); Syntypes: Venezuela, Caracas, April 1842, *Linden 620* (W-R51649); Agua de Obispo, prov. Truxillo, 7000 feet, May, *Linden 620* (BR0000013083625); prov. Merida, *Linden 620* (W-R17081); no thorough locality from Venezuela, *Linden 620* (W-R51295).

#### Note.

**Figure 2.**
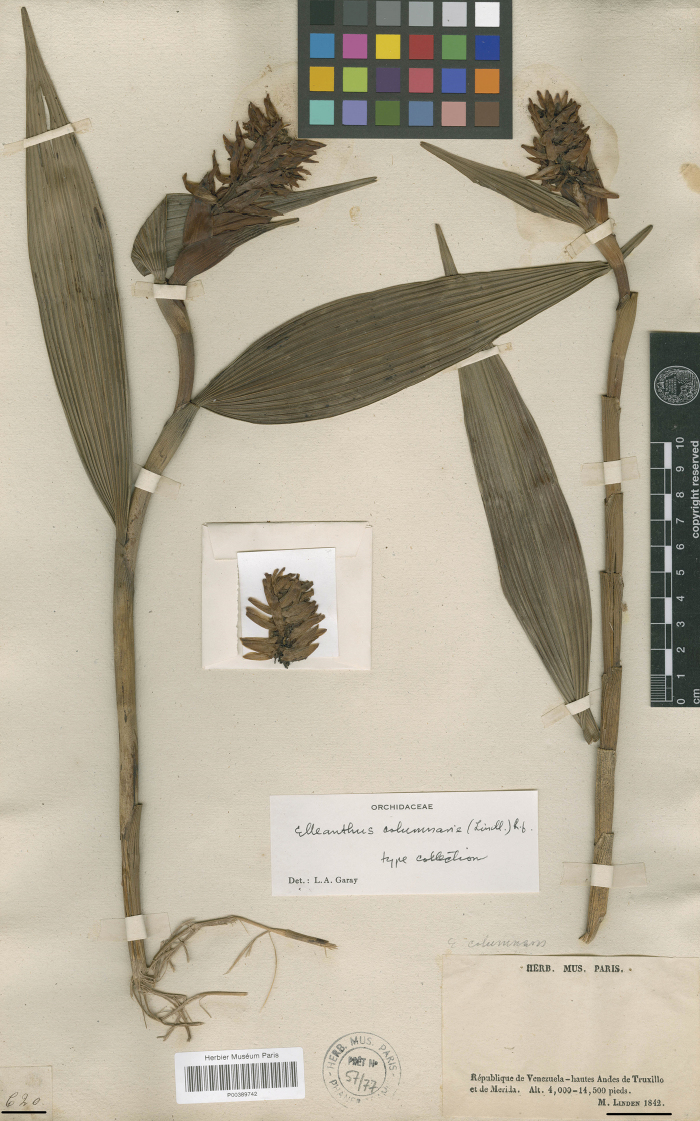
*Evelynacolumnaris* Lindl. Specimen *Linden 620* at the Museum National d’Histoire Naturelle, Paris (P00389742) designated here as lectotype (CC BY 4.0; http://coldb.mnhn.fr/catalognumber/mnhn/p/p00389742).

In the protologue of *Evelynacolumnaris*, Lindley (1846:11) cites, as the type, the collection named *Linden 620* from ‘Agua de Obispo and Sierra Nevada, at the height of 9000 feet, May to August’. We found that the collection labelled *Linden 620* actually consists of six specimens deposited at W, P, BR and K herbaria. These specimens were collected at four distinct Venezuelan localities: Caracas, Agua de Obispo prov. Truxillo (=Trujillo), prov. Merida, the high Andes of Truxillo and Merida and the last one with no precise location. Thus, these specimens should be treated as syntypes (Art. 9.4 Shenzhen Code), with the Parisian one serving as the lectotype (Fig. 2). It contains not only vegetative parts, which are very well preserved, but also the inflorescence with many flowers. It was collected in the high Andes mountains in the Provinces of Trujillo and Merida, which corresponds to the protologue. A sheet kept at Kew bears a drawing of flower segments (a dorsal sepal, a lateral sepal, a petal and a lip) and a gynostemium, which were made, based on the original material.

### Elleanthus coriifolius

Taxon classificationPlantaeAsparagalesOrchidaceae

(Rchb.f ex Linden) Rchb.f., Annales Botanices Systematicae 6: 478. 1861.

3A24C557-4E25-5193-BB76-0E375389DA73

Basionym: Evelynacoriifolia Rchb. f. *ex* Linden, Botanische Zeitung (Berlin) 10: 710. 1852. Type: Colombia, “Neu Granada”, *Linden 1272*; Lectotype (designated here): W-R (W-R51672).

#### Note.

**Figure 3.**
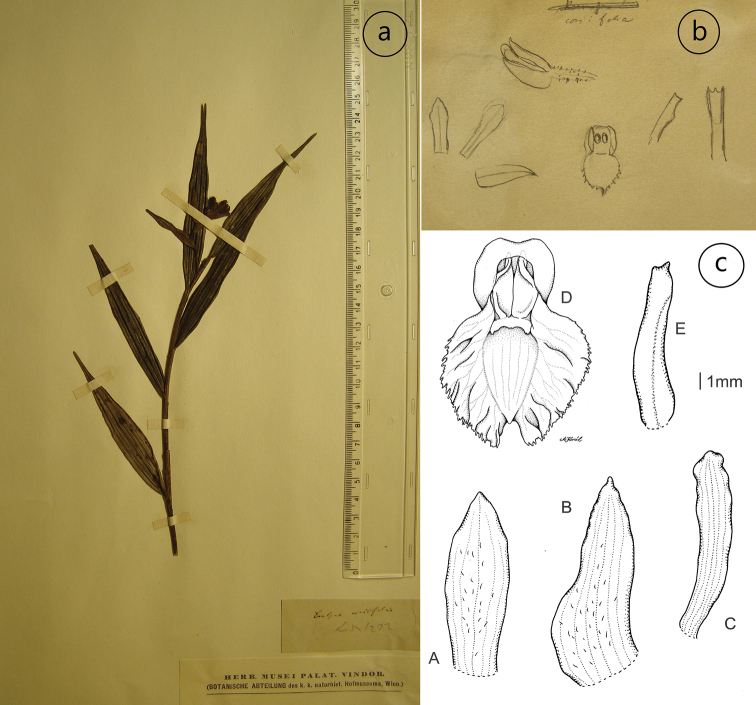
Evelynacoriifolia Rchb. f. *ex* Linden **a** specimen *Linden 1272* at the Naturhistorisches Museum, Vienna (W-R51672) designated here as lectotype **b** Reichenbach’s drawing **c** based on Szlachetko’s drawing of the original material (A. Król): A – dorsal sepal, B – lateral sepal, C – petals, D – lip, E – gynostemium.

In the protologue of *Evelynacoriifolia* Rchb.f., the author indicates the gathering *Linden 1272* as the type. We found only one specimen of this collection deposited in the Naturhistorisches Museum in Vienna (Fig. 3a). This specimen has no type designation, but it is labelled with the same collector as in the protologue. We analysed the vegetative and floral features and compared them with diagnosis. It is noteworthy that our analytical drawing (Fig. 3c) is somewhat different from details depicted by Reichenbach (Fig. 3b). In our opinion, the only explanation is that Reichenbach prepared his sketches, based on premature flower and this this is clearly visible on his illustration of the flower (upper part of the sketch), which is narrowly tubular. His presentation of the lip clearly suggests that the apical part of the lip was not fully pressed and corpuscles are undeveloped.

In addition, in Kew, there is a sheet which bears a drawing made on the basis of a specimen from Vienna. It is marked as a type as well; however, it was not made by the author of the species. According to the Code of Nomenclature (Turland et al. 2018) this drawing cannot be treated as part of the original material.

### Elleanthus ensatus

Taxon classificationPlantaeAsparagalesOrchidaceae

(Lindl.) Rchb. f., Annales Botanices Systematicae 6: 482. 1861.

3B623AB3-F526-5B84-9499-F3518D79EE0B

Basionym: Evelynaensata Lindl., Orchidaceae Lindenianae 11–12. no. 64. 1846. Type: Venezuela, Merida, “Sierra Nevada, at the height of 8000 feet [2438 m], August”, *Linden 664*; Lectotype (designated here): P (P00389702); isolectotypes: W (W-R51384), W (W-R51392), W-R-drawing (W-R30233).

#### Note.

**Figure 4.**
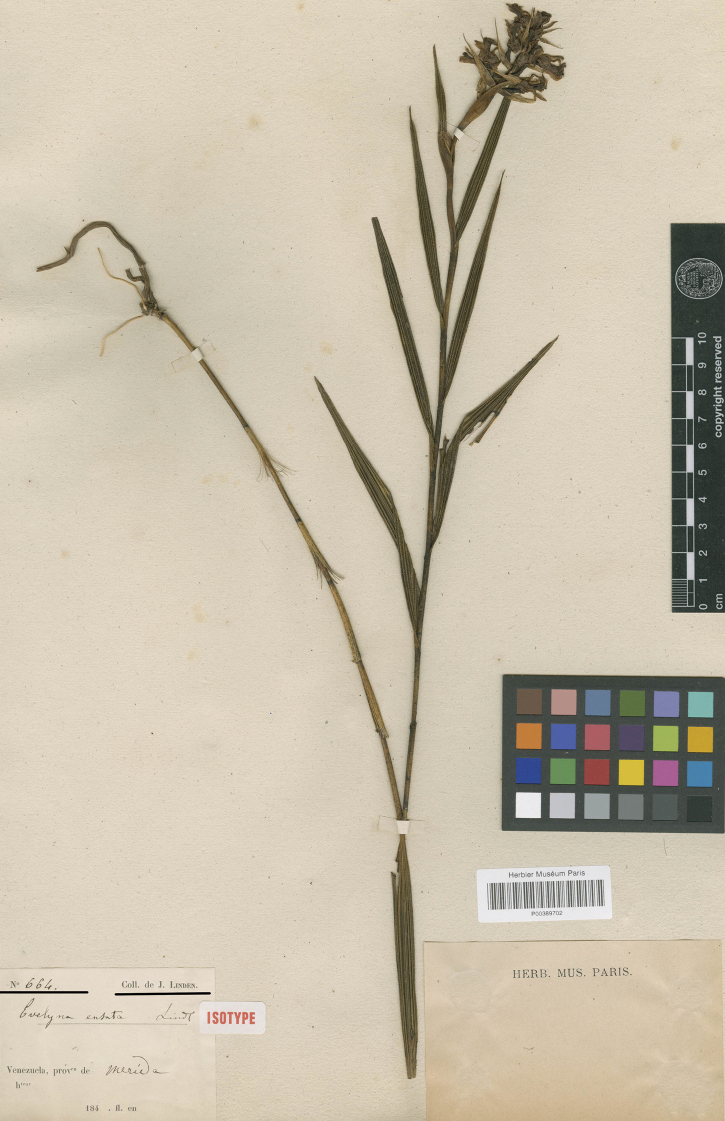
*Evelynaensata* Lindl. Specimen *Linden 664* at the Museum National d’Histoire Naturelle, Paris (P00389702), designated here as lectotype (CC BY 4.0; http://coldb.mnhn.fr/catalognumber/mnhn/p/p00389702).

In the protologue of *Evelynaensata* Lind., the author indicates in a short note the gathering of *Linden 664* as the type. We found that this collection actually consists of three specimens deposited in two institutions: the Naturhistorisches Museum in Vienna and the Muséum National d’Histoire Naturelle in Paris. The Vienna specimens have no annotation on their status, but they have a designation of the collection number *Linden 664*; the specimen stored in P is labelled as the type. We have analysed and compared all of them with the diagnosis. We analysed not only vegetative features, but also we compared the floral structures. These specimens have all the features of *Evelynaensata* and one of them was selected as the lectotype (Fig. 4).

There is a Reichenbach drawing kept at W (W-R30233), labelled *E.ensata* and numbered 644. In the protologue, Lindley (1846: 12) indicates a gathering *Linden 664*. However, drawing deposits in Vienna Herbarium definitely correspond with Lindley’s species. Therefore, we can assumed that this is probably an error.

### Elleanthus flavescens

Taxon classificationPlantaeAsparagalesOrchidaceae

(Lindl.) Rchb.f., Annales Botanices Systematicae 6: 479. 1862.

58AA7B81-244A-5E7F-93F0-D5B700BD584E

Basionym: Evelynaflavescens Lindl., Orchidaceae Lindenianae 10, no. 59. 1846. Type: Venezuela, Trujillo “between Humucoro-Bajo and the Agua de Obispo, in the Province of Truxillo, at the height of 7000 feet [2133 m], May”, *Linden 625*; Lectotype (designated here): K-L; isolectotypes: P (P00389695), W-R (W-R51662, W-R51664, W-R30242 [drawing]), BR (BR0000013083618).

#### Note.

In the short note following the protologue, Lindley (1846) mentions a gathering which may refer to this species: *Linden 625*. Dodson and Luer (2010) selected the specimen kept in the Kew Herbarium as the holotype, but according to Arts. 9.11 and 9.12 of the Code of Nomenclature, their designation did not constitute a typification (Turland et al. 2018). This specimen could be treated as the lectotype.

During our studies, we found five more specimens which are labelled *Linden 625*. Three of them are deposited in the Naturhistorisches Museum in Vienna, one in the Muséum National d’Histoire Naturelle in Paris and one in Meise Botanic Garden in Belgium. The specimens from Vienna have no type annotation, but do mention the collection as *Linden 625*. We analysed the vegetative and floral characteristics and compared them with the diagnosis; two of them (W-R51662 and W-R51664) represented *Elleanthusflavescens*. The third one (W-R30242) is a drawing which is likely based on the type material as the same collector and number are recorded on it as in the protologue.

The specimen deposited in the Paris Herbarium (P00389695) was labelled by Garay as *Elleanthusfurfuraceus* (Lindl.) Rchb.f. However, we are of the opinion that it is a part of the type material of *Elleanthusflavescens*. This specimen has lanceolate, acuminate leaves, an oblong, cylindrical inflorescence which is loose at the base, floral bracts that are shorter than the flowers and a pair of ovate corpuscules at the base of the lip with a strongly thickened transverse ridge, just like *E.flavescens*. It also mentions the number *Linden 625*.

*Elleanthusflavescens* is more similar to *E.aurantiacus* than *E.furfuraceus*. Some authors, such as Foldats (1969) and Luteyn (1999), treat this species as a synonym of *E.aurantiacus*. However, it is distinguished from the latter by smaller flowers, less cone-shaped inflorescences and a decidedly thicker transverse callus (Dunsterville and Garay 1966, 1979; Dodson and Luer 2010).

### Elleanthus furfuraceus

Taxon classificationPlantaeAsparagalesOrchidaceae

(Lindl.) Rchb.f., Annales Botanices Systematicae 6: 480. 1861.

2050F2FC-108F-593E-A517-CDFC629B8897

Basionym: Evelynafurfuracea Lindl., Orchidaceae Lindenianae 12. no. 65. 1846. Type: Venezuela, Trujillo “Agua de Obispo, at the height of 9000 feet [2743 m], May”, *Linden 627*; Lectotype (designated here): P (P00389698); isolectotypes: P (P00389697), BR (BR0000013083588), W-R (W-R17083); Syntype: Venezuela, Merida “forest of Merida, at the height of 5500 feet [1676 m], June”, *Linden 619* (unknown location).

#### Note.

**Figure 5.**
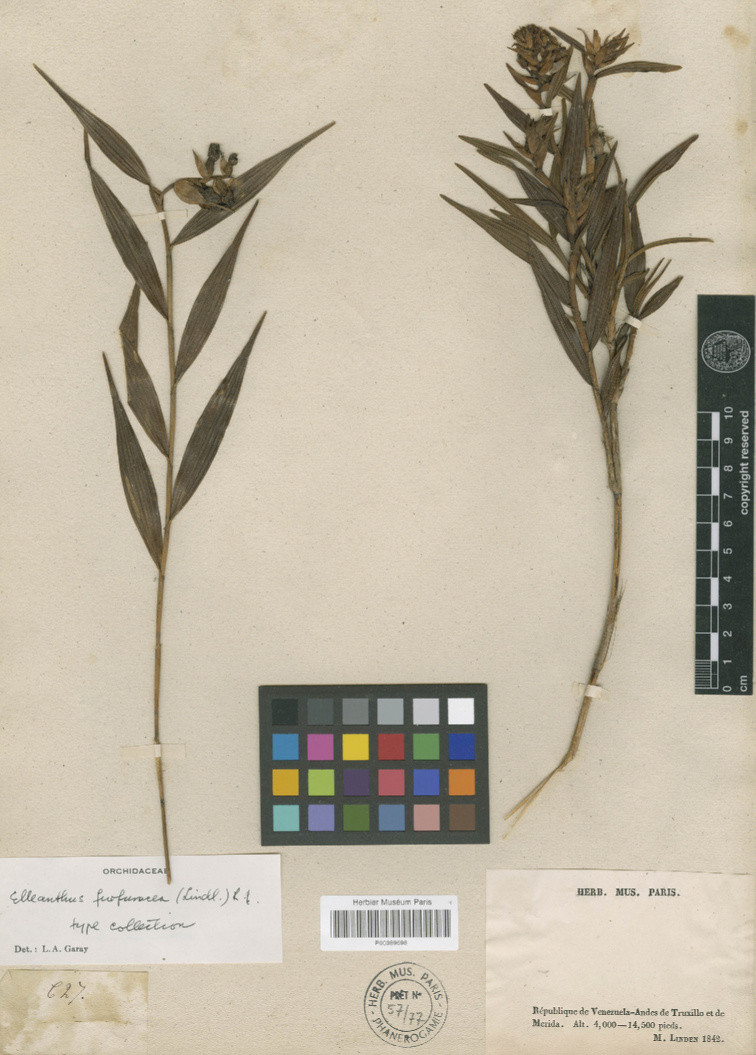
*Evelynafurfuracea* Lindl. Specimen *Linden 627* at the Museum National d’Histoire Naturelle, Paris (P00389698) designated here as lectotype (CC BY 4.0; http://coldb.mnhn.fr/catalognumber/mnhn/p/p00389698).

In describing *Evelynafurfuracea*, Lindley (1846: 12) cited two Linden collections: *619* and *627*. Unfortunately, we were unable to locate *Linden 619*. However, collection *Linden 627* is stored in the Muséum National d’Histoire Naturelle in Paris (two specimens), in Meise Botanic Garden (one specimen) and in the Naturhistorisches Museum in Vienna (one specimen). However, only the specimens in P and BR are marked as the type collection and only one of them is a complete specimen (P00389698) (Fig. 5). It can be characterised by lanceolate, acuminate and coriaceous leaves; terminal, laxly to subdensely flowered (with few to several) inflorescences; and subrounded-ovate, acute bracts. This specimen has also a lip obovate to suborbicular in outline, concave, unlobed and retuse in the front and saccate at the base with two large, well-separated, ellipsoid/ovoid calli. Therefore, it has been selected as the lectotype (Fig. 5).

### Elleanthus kermesinus

Taxon classificationPlantaeAsparagalesOrchidaceae

(Lindl.) Rchb.f., Annales Botanices Systematicae 6: 478. 1862.

C1246525-D406-57F1-BE14-64FF46502366

Basionym: Evelynakermesina Lindl., Orchidaceae Lindenianae 11. no 61. 1846. Type: Venezuela, Tolima, Mariquita “from the forests of Tolima in the Province of Mariquita at the height of 9000 feet [2743 m], January”, *Linden 1276*; Lectotype (designated here): P (P00419576); isolectotype: BR (BR0000013083366).

#### Note.

**Figure 6.**
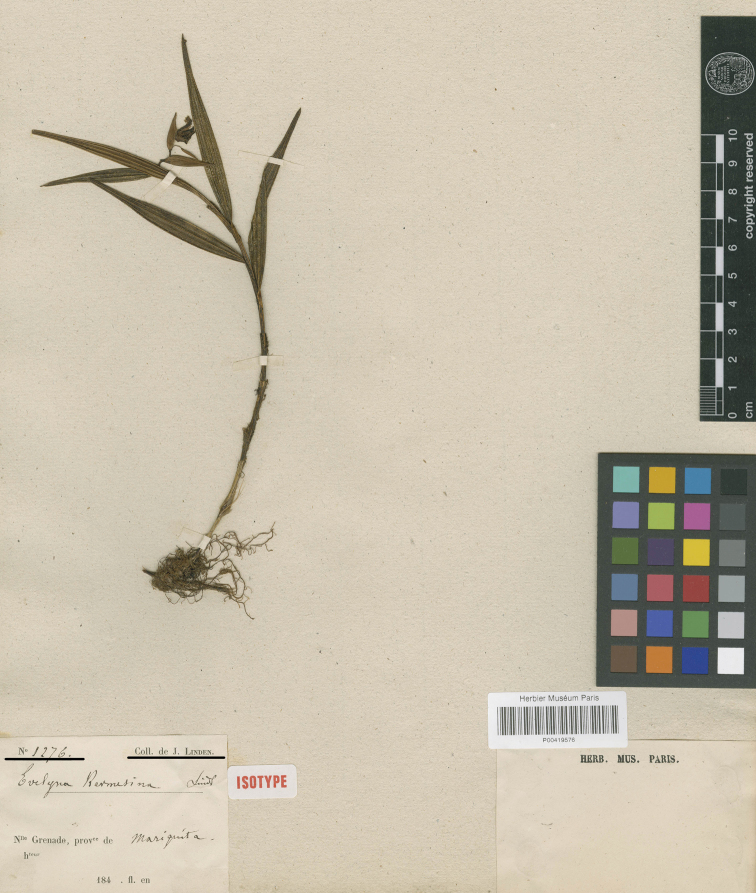
*Evelynakermesina* Lindl. Specimen *Linden 1276* at the Museum National d’Histoire Naturelle, Paris (P00419576) designated here as lectotype (CC BY 4.0; http://coldb.mnhn.fr/catalognumber/mnhn/p/p00419576).

In the short note following the protologue, Lindley (1846: 11) cited the collection *Linden 1276*. We have found two specimens corresponding to the original description. All of them bear *Linden 1276* and were labelled as *Evelynakermesina* Lindl. and designated as the type. These are deposited in the following Herbaria: the Muséum National d’Histoire Naturelle and Meise Botanic Garden. However, in the Botanic Garden in Kew, there was found a drawing based on type material, but it was not made by the author of species. This sheet embraces particular segments of flowers (a dorsal sepal, a lateral sepal, a petal and a lip), a floral bract and a gynostemium. The best preserved specimen, kept in the Paris Herbarium (P00419576), contains not only vegetative parts, but also an inflorescence with a flower (Fig. 6). The specimen has linear/lanceolate, mucronate and coriaceous leaves, a fractiflex inflorescence and two small, oval calli on the base of the lip, which displays transverse thickening. According to Arts. 9.11 and 9.12 (Turland et al. 2018), this specimen could be treated as the lectotype. The specimen from Meise Botanic Garden (BR0000013083366) is a sterile plant without flowers.

### Elleanthus lupulinus

Taxon classificationPlantaeAsparagalesOrchidaceae

(Lindl.) Rchb.f., Annales Botanices Systematicae 6: 483. 1861.

7578A7C3-AAA9-5BEE-81BE-839CE2B71323

Basionym: Evelynalupulinus Lindl., Orchidaceae Lindenianae 11. no. 63. 1846. Type: Venezuela, Merida “plant from the vicinity of the Paramo of the Sierra Nevada, at the height of 10 000 feet [3038 m], August”, *Linden 642*; Lectotype (designated by Garay 1978: 88): K-L; isolectotypes: P (P00389658), W-R (W-R17080, W-R51365), MO (MO1109600).

#### Note.

Dodson and Luer (2010) selected a specimen from the Kew Herbarium as the holotype. This action is against the rules of the Code of Botanical Nomenclature. In the protologue, Lindley (1846) did not indicate where the type collection was deposited. In accordance with Arts. 9.11 and 9.12 of the ICN (Turland et al. 2018), if the plant name was published without indicating a holotype, a lectotype can be selected. We found that the gathering of *Linden 642* actually consists of five specimens deposited at K, P, W and MO. All of these specimens are labelled as the type and a comparison of the features against the original description of the species reveals that they correspond to *Evelynalupulina* Lindl. In such a situation, the lectotype may be designated from amongst these specimens. However, Garay (1978), in Flora of Ecuador, used the term type for the K specimen, while it can serve as the lectotype. According to Art. 9.10 of the Code of Nomenclature (Turland et al. 2018), Garay unknowingly designated a lectotype. A misused term may be corrected because this case meets the requirements of Art. 7.11 (Turland et al. 2018).

## Supplementary Material

XML Treatment for Elleanthus bractescens

XML Treatment for Elleanthus columnaris

XML Treatment for Elleanthus coriifolius

XML Treatment for Elleanthus ensatus

XML Treatment for Elleanthus flavescens

XML Treatment for Elleanthus furfuraceus

XML Treatment for Elleanthus kermesinus

XML Treatment for Elleanthus lupulinus
